# Cancer Stem Cells in Hepatocellular Carcinoma: Intrinsic and Extrinsic Molecular Mechanisms in Stemness Regulation

**DOI:** 10.3390/ijms232012327

**Published:** 2022-10-14

**Authors:** Xiaona Fang, Qian Yan, Shan Liu, Xin-Yuan Guan

**Affiliations:** 1 Department of Clinical Oncology, The University of Hong Kong-Shenzhen Hospital, Shenzhen 518000, China; 2 Department of Clinical Oncology, The University of Hong Kong, Hong Kong 999077, China; 3 State Key Laboratory for Liver Research, The University of Hong Kong, Hong Kong 999077, China; 4 Guangdong Institute of Gastroenterology, Guangdong Provincial Key Laboratory of Colorectal and Pelvic Floor Diseases, The Sixth Affiliated Hospital, Sun Yat-sen University, Guangzhou 510000, China; 5 MOE Key Laboratory of Tumor Molecular Biology, Jinan University, Guangzhou 510000, China; 6 Advanced Nuclear Energy and Nuclear Technology Research Center, Advanced Energy Science and Technology Guangdong Laboratory, Huizhou 516000, China

**Keywords:** cancer stem cells, hepatocellular carcinoma, cell surface markers, heterogeneity, stroma cells, immune cells, CSCs-targeting therapies

## Abstract

Hepatocellular carcinoma (HCC) remains the most predominant type of liver cancer with an extremely poor prognosis due to its late diagnosis and high recurrence rate. One of the culprits for HCC recurrence and metastasis is the existence of cancer stem cells (CSCs), which are a small subset of cancer cells possessing robust stem cell properties within tumors. CSCs play crucial roles in tumor heterogeneity constitution, tumorigenesis, tumor relapse, metastasis, and resistance to anti-cancer therapies. Elucidation of how these CSCs maintain their stemness features is essential for the development of CSCs-based therapy. In this review, we summarize the present knowledge of intrinsic molecules and signaling pathways involved in hepatic CSCs, especially the CSC surface markers and associated signaling in regulating the stemness characteristics and the heterogeneous subpopulations within the CSC pool. In addition, we recapitulate the effects of crucial extrinsic cellular components in the tumor microenvironment, including stromal cells and immune cells, on the modulation of hepatic CSCs. Finally, we synopsize the currently valuable CSCs-targeted therapy strategies based on intervention in these intrinsic and extrinsic molecular mechanisms, in the hope of shedding light on better clinical management of HCC patients.

## 1. Introduction

According to the Global Cancer Statistics in 2020, hepatocellular carcinoma (HCC) constitutes about 80% of primary liver malignancies, which rank as the sixth most common cancer and the third leading cause of cancer-related death worldwide [1]. The incidence rate and mortality of HCC are continuing to rise, posing a great challenge to global health [2]. Due to the absence of clinical symptoms during its early stages, most patients are in the advanced stage at the first diagnosis and develop liver or distal metastasis. Even for the minority of patients with early-stage HCC who receive surgical resection, the recurrence rate reaches around 70% after surgery [3]. The estimated 5-year survival time in patients with advanced HCC is only 3 to 13 months after systematic therapies [4]. Overall, the therapeutic efficacy and prognosis in HCC patients are extremely poor. It has been documented at single-cell resolution that HCC is a biologically complex malignancy with highly heterogenous genetic and cellular dysregulations and a complicated tumor microenvironment [5,6,7,8,9,10]. Accumulating evidence has demonstrated that cancer stem cells (CSCs), a small group of cells harboring the capacities to self-renew and initiate tumor, are notably responsible for tumor heterogeneity, recurrence, metastasis, as well as resistance to chemotherapy [11]. Therefore, elucidation of how these CSCs maintain their stem cell-like features is essential for the development of CSCs-based therapy and durable treatment of HCC patients. Among the various treatment strategies for HCC patients, CSCs-targeted therapy would be a very promising regimen.

Within the tumor bulk, hepatic CSCs are distinguished by their expression of various stemness-related markers and activation of stemness-associated regulatory signaling pathways that contribute to maintain their stem cell traits. In the surrounding intricate tumor microenvironment (TME), the cellular components, especially stroma cells and immune cells, play critical roles in the stemness regulation of CSCs. Further investigation into the intrinsic and extrinsic molecular mechanisms triggering the biological properties of CSCs could facilitate the advancement of CSCs-directed therapy. In this review, we enumerate the well-recognized and newly discovered CSC surface markers and their representative signaling pathways involved in stemness regulation. We also recapitulate the role of stroma cells and immune cells in driving the tumor-initiating abilities of CSCs, thus providing insights for CSCs-based therapies from these perspectives. The currently valuable CSCs-targeted therapy strategies based on these mechanisms are also summarized.

## 2. Intrinsic Molecules and Involved Signaling Pathways

### 2.1. Cell Surface Markers of Hepatic CSCs

The past three decades have witnessed a rich development in excavating important CSCs surface markers. These well-known and newly identified surface markers and associated signaling pathways are summarized in Table 1.

CD133, also known as prominin 1 (PROM1), is considered the most compelling cell surface marker for the identification of hepatic CSCs. Substantial evidence has elucidated the stemness-associated features of CD133+ cells, including self-renewal, chemoradiotherapy resistance, tumorigenicity, and metastasis. The intrinsic activations of multiple signaling pathways were found to be involved in regulating CD133+ CSCs behavior, such as the Wnt/β-catenin signaling [12,13,14,15], IL-6/STAT3 signaling [16], TGF-β pathway [17], AKT/PKB pathway [18], IL-8/MAPK signaling [19], TLR4/NANOG and STAT3 signaling [20], STAT3/SOX4 signaling [21], MEK/ERK signaling [22], and ANXA3/JNK signaling [23]. In addition to CD133, CD44 and the epithelial cell adhesion molecule (EpCAM) are also well-characterized CSC surface markers. CD44+ CSCs showed enhanced sphere formation and migration abilities through upregulation of AKT/GSK-3β/β-catenin signaling [24]. In addition, TGF-β, JAK/STAT, and IL-6/STAT3 signaling pathways contributed prominently to the stemness regulation of CD44+ cells, resulting in their vital roles in tumor initiation and growth [25,26,27]. Regarding EpCAM+ CSCs, various pathways were invariably hijacked to maintain stemness characteristics. For instance, the crosstalk between TNF-α/NF-κB and IL-6/STAT3 signaling was reported to be critical for EpCAM+ CSCs expansion in HCC [28]. Likewise, Wnt/β-catenin signaling was found to be enriched in the EpCAM+ cell population, which showed augmented self-renewal and differentiation abilities [29]. Accumulating studies have revealed that CD90+ HCC cells exhibited self-renewal, pro-tumorigenicity and aggressive phenotypes [30] with the activation of AKT/EphA2 signaling [31], IL-6/JAK2 signaling [32], AKT and mTOR signaling [33] as well as MAP3K8 signaling [34]. Moreover, CD13 is also a well-explored stem cell surface marker in HCC. The molecular mechanisms involved in stemness regulation of CD13+ cells consisted of the activation of Wnt/β-catenin signaling, NF-kB signaling and YAP1 signaling [35,36,37]. Interestingly, Li Sun et al. reported that aerobic metabolism of tyrosine signaling endowed CD13+ CSCs with increased organoid formation and resistance to chemotherapy [38]. Similar to CD133+ and CD44+ cells, IL-6/STAT3 signaling also contributed largely to the improved stemness properties and tumorigenicity of CD24+ CSCs [28]. Besides, CD24+ cells also employed STAT3-mediated NANOG regulation [39] and iNOS-mediated TACE/ADAM17/NOTCH signaling [40] to fulfill their stem cell traits. Cell surface protein CD47 has been reported to be correlated with self-renewal, tumor initiation, and metastasis in HCC through upregulating CTSS/PAR2 signaling [41]. Another potential CSC marker, CD54, also known as intercellular adhesion molecule 1 (ICAM1), was demonstrated to confer self-renewal and tumor malignant transformation capacities to HCC cells [42,43]. As the most common and functional stemness-associated pathway, β-catenin signaling was verified to be enriched in leucine rich repeat containing G protein-coupled receptor 5 (LGR5) positive [44,45,46,47] and OV6+ liver cancer-initiating cells to sustain their CSCs identities [48,49]. CD34+ HCC cells displayed stem cell features such as self-renewal and tumor initiation via upregulation of various pluripotency drivers such as OCT4, SOX2, NAONG, KLF4 and c-MYC [50,51,52]. In addition, a recent study found that the calcium channel α2δ1 subunit could be a targetable hepatic tumor-initiating cell marker [53]. The α2δ1+ liver CSCs exhibited enhanced self-renewal and tumorigenicity abilities through ERK1/2 phosphorylation compared to the α2δ1- subset [54,55]. Another cell surface protein delta-like 1 protein (DLK1), endowed HCC cells with stronger ability of self-renewal, chemoresistance and tumorigenicity compared to DLK1− cells [56,57,58].

Apart from early identified CSC markers, other potential surface markers of CSCs in HCC were also discovered and characterized. A recent study revealed that CD73+ HCC cells sustained robust CSC features, including self-renewal ability, expression of stemness-related genes and drug resistance via harnessing AKT signaling and SOX9 expression [59]. CD206, expressed on the cell surface, was also found to be a potential hepatic CSC marker that was co-expressed in spheroids with CD44 and several classic pluripotency transcription factors, including OCT4, SOX2, NANOG and c-MYC [60]. Collectively, Wnt/β-catenin signaling and IL-6/STAT3 signaling are the top two pathways regulating the stem cell features of diverse marker-positive CSCs, including CD133, EpCAM, CD24 and LGR5, indicating that targeting these signaling pathways may be a promising strategy to eliminate CSCs and reverse drug resistance.

**Table 1:** Surface markers of hepatic CSCs.

CSC Markers	Source of Identification	Phenotypes	Signaling Involved in CSCs	Clinical Predictive Value	Refs.
CD133	Cell lines, Primary tissues	Self-renewal, Tumorigenicity, Chemoresistance, Invasiveness, Cell proliferation, Radioresistance	Wnt/β-catenin signaling, IL-6/STAT3 signaling, IL-8/MAPK signaling, AKT/PKB pathway, MEK/ERK signaling, STAT3/SOX4 signaling, ANXA3/JNK signaling, TGF-β gaining, TLR4/NANOG and STAT3 signaling	Diagnostic, Therapeutic, Prognostic	[12,13,14,15,16,17,18,19,20,21,22,23,36,61,62,63]
CD44	Cell lines, Primary tissues	Sphere formation, Tumorigenicity, Targeted drug resistance, TGF-β-mediated mesenchymal phenotype, EMT	TGF-β signaling, JAK/STAT signaling, IL-6/STAT3 signaling, AKT/GSK-3β/β-catenin signaling	Therapeutic, Prognostic	[24,25,26,27,64,65,66,67]
EpCAM	Cell lines, Primary tissues	Self-renewal, Differentiation, Drug resistance	Wnt/β-catenin signaling, TNF-α/NF-κB, IL-6/STAT3 signaling	Diagnostic, Therapeutic, Prognostic	[28,29,68]
CD90	Cell lines, Primary tissues	Sphere formation, Tumorigenicity, Metastasis, Cell migration and invasion, Cell proliferation, Chemoresistance	AKT/EphA2 signaling, IL-6/JAK2 signaling, AKT and mTOR signaling, MAP3K8 signaling	Diagnostic, Therapeutic, Prognostic	[30,31,32,33,34,69,70]
CD13	Cell lines, Primary tissues	Sphere formation, Tumorigenicity, Chemoresistance, Angiogenesis, ROS-induced DNA damage	Wnt/β-catenin signaling, YAP1 signaling, NF-kB signaling, Aerobic metabolism of tyrosine signaling	Therapeutic, Prognostic	[35,36,37,38,71]
CD24	Cell lines, Primary tissues	Sphere formation, Stemness gene expression, Tumorigenicity, Chemoresistance, Cell migration and invasion	IL-6/STAT3 signaling, STAT3-mediated NANOG regulation, iNOS-mediated TACE-ADAM17-NOTCH signaling	Therapeutic, Prognostic	[28,39,40,65,72]
CD47	Cell lines, Primary tissues	Tumor initiation, Self-renewal, Tumorigenicity, Chemoresistance, Metastasis	CTSS–PAR2 signaling	Therapeutic, Prognostic	[41]
CD54/ ICAM1	Cell lines, Primary tissues	Sphere formation, Tumorigenicity, Metastasis	NANOG dysregulation	Therapeutic, Prognostic	[42,43]
LGR5	Cell lines	Sphere formation, Tumorigenicity, Organoid initiation, Tumor growth, Drug resistance	Wnt/β-catenin signaling	Therapeutic, Prognostic	[44,45,46,47]
OV6	Cell lines, Primary tissues	Tumorigenicity, Chemoresistance, Invasion, Metastasis	β-catenin signaling	Therapeutic, Prognostic	[48,49,73]
CD34	Cell lines	Self-renewal, Tumorigenicity	OCT4, SOX2, NAONG, KLF4, c-MYC upregulation	Prognostic	[50,51,52]
Calcium channel α2δ1 subunit	Cell lines	Self-renewal, Tumorigenicity	ERK1/2 phosphorylation	Therapeutic	[53,54,55]
DLK1^m^	Cell lines	Chemoresistance, Colony formation, Spheroid formation, Tumorigenicity	Not reported	Therapeutic	[56,57,58]
CD73	Cell lines	Sphere formation, Lenvatinib resistance, Stemness gene expression	AKT signaling, SOX9 upregulation	Therapeutic, Prognostic	[59]
CD206	Cell lines	Cell migration and invasion	Not reported	Prognostic	[60]

### 2.2. Heterogeneous Patterns of Hepatic CSC Surface Markers and Phenotypes

**Figure 1:**
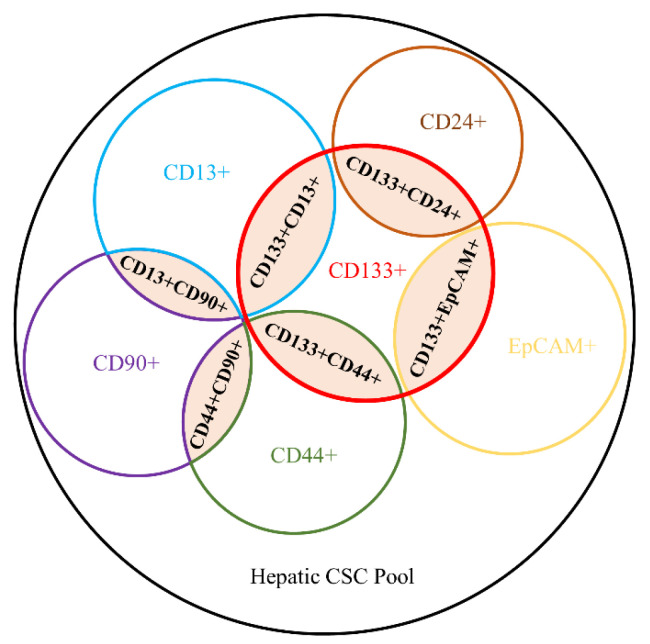
A cartoon showing the heterogeneous nature of CSCs in HCC. Illustrated are six subsets with individual surface marker expressions harboring distinct stemness features inside the hepatic CSC pool. CSCs with a combined expression of surface markers (shaded) possessed more vital tumor-initiating capacities than unique marker-expressed CSCs.

Although marker-positive cancer cells were defined as CSCs, these markers are not all concomitantly expressed in the same subpopulation. It is well known that heterogeneity exists not only in tumors but also in hepatic CSCs, which may be organized in a hierarchical relationship [74]. Marker-positive cancer cells are not randomly distributed but spatially heterogeneous, and hepatic CSC subpopulations showed vast heterogeneity biologically and phenotypically [10]. Furthermore, the hepatic CSC subgroup expressing certain CSC markers may not co-occur in all HCC tumors. Different CSC subclusters may exhibit distinct expression patterns of CSC markers and display phenotypically diverse features and functions. Yamashita et al. uncovered that EpCAM+ and CD90+ CSCs represented distinct CSC subpopulations in primary HCC tissues and showed discrete features in terms of cell morphology, tumorigenicity and metastasis propensity [75]. Different CSC markers, including CD133, EpCAM and CD24, defined distinct CSC subclusters, which exhibited different self-renewal potentials [10]. Moreover, the combined expression of certain individual hepatic CSC markers has also fueled CSCs with stronger stemness and tumorigenic characteristics. For example, CD133+CD44+ CSCs showed enhanced abilities of sphere formation, stemness-related gene expression, tumorigenesis and chemoresistance compared with CD133+CD44− cells [76]. CD90+CD44+ CSCs were evidenced with more aggressive and pro-metastatic phenotypes than CD90+CD44− counterparts [77]. Similarly, CD133+EpCAM+ CSCs exhibited remarkable and greater tumor-initiating power compared to CD133+ cells [29]. The CD133+CD13+ fraction harbored a stronger self-renewal capacity and high resistance to chemotherapy compared to the CD133+CD13− subset [78] and an enhanced self-renewal and tumorigenicity compared to the CD133-CD13− subset via the lncTCF7-mediated Wnt signaling pathway [15]. Interestingly, it was revealed that the self-renewal ability and radiotherapy-resistant potential of CD13+CD90− cells were significantly superior to CD13-CD90+ cells [78]. Another study uncovered the presence of CD133+CD24+ cells in HCC tumors showing stemness features both *in vitro* and *in vivo*, which were not observed in CD133-CD24- cells [40]. The heterogeneous nature of CSCs in HCC is depicted in Figure 1. These heterogeneous expression patterns also indicate that even in one marker-positive CSC, there may exist distinct subclusters, which were demonstrated by a recent study highlighting the heterogeneity of CD133+ HCC cells [79]. Given the diverse expression of surface markers and distinct stemness traits of CSC subpopulations, the potential hierarchical structure of hepatic CSCs is still far from clear and needs to be further studied. Targeting more than one hepatic CSC marker may be a more effective way to eliminate CSCs and be beneficial for HCC patients.

## 3. Extrinsic Cellular Components in the Tumor Microenvironment

While intrinsic molecules and signaling pathways in CSCs have been a principal focus of previous studies, how the TME may impact CSC behaviors has been less deciphered. Although little is known about the CSC niche in cancer, it is supposed that CSCs reside in a friendly environment that supports their stemness and maintains the CSC subpopulation [80]. An extensive body of studies has revealed that the various cell types and factors in the tumor ecosystem were indispensable for the formation of the CSC niche, indicating that CSCs-based therapy only targeting CSCs signaling may be insufficient to ablate CSCs [81]. Further study of the CSCs-supportive TME signals may facilitate the development of novel regimens to eradicate CSCs and achieve sustained anti-cancer efficacy. The most critical cellular components essential for the CSC niche are stroma cells such as cancer-associated fibroblasts (CAFs), adipocytes, endothelial cells (ECs) and various kinds of immune cells, including tumor-associated macrophages (TAMs), tumor-associated neutrophils (TANs), T cells, B cells, natural killer (NK), dendritic cells (DCs) and myeloid-derived suppressor cells (MDSCs) [82]. Below, we discuss and summarize how these neighboring cell subsets affect hepatic CSCs and mediate their stemness and malignant properties.

Increasing studies have revealed that CAFs played critical roles in HCC development and aggressiveness. The conditional medium from CAFs could fuel cancer stemness traits, including the self-renewal of CSCs, expression of stemness-associated markers and oncofetal proteins, metastasis, and drug resistance to chemotherapy, suggesting indirect communications between CAFs and cancer cells. It was reported that CAFs mediated the stemness properties of HCC cells through the secretion of the hepatocyte growth factor (HGF), which stimulated MET/FRA1/HEY1 signaling in hepatic CSCs [83]. Similarly, IL-6 produced by CAFs was evidenced to endow tumor-initiating characteristics to HCC cells by the enrichment of STAT3/Notch signaling [84]. Increased excretion of IL-6 and IL-8 from CAFs regulated by HCC-derived miR-1247-3p was also demonstrated to promote stemness and metastasis in HCC [85]. A study by Li et al. further reported that CAFs-derived HGF and IL-6 contributed drastically to the self-renewal, tumorigenicity, metastasis and chemoresistance of CD24+ liver CSCs via the phosphorylation of the STAT3 signaling pathway [86]. Besides, Sun et al. uncovered that CAFs-secreted cartilage oligomeric matrix protein (COMP) could ameliorate the self-renewal and epithelial-mesenchymal transition (EMT) function of HCC cells through the induction of MEK/ERK and PI3K/AKT signaling [87,88]. Surprisingly, autophagy was found to be associated with HCC stemness by CAFs-induced autophagic flux [89]. In addition, the Song group indicated that cardiotrophin-like cytokine factor 1 (CLCF1), derived from CAFs, could facilitate stem-like characteristics of HCC cells by STAT3/CXCL6/E2F1 and STAT3/TGF-β/p38 signaling axes [90]. Interestingly, HCC-produced chemokine (C-X-C motif) ligand 6 (CXCL6) and TGF-β could in turn activate ERK1/2 signaling in CAFs and promote CLCF1 expression and secretion and form a positive feedback loop [90]. Furthermore, a recent study revealed that the stemness capacities of HCC cells could be potentiated by CAFs-derived TGF-β1 through P85a/AKT/TBX3 signaling [91]. A newly identified secreted protein by CAFs in HCC, follistatin-like 1 (FSTL1), was also found to have participated in stemness modulation and malignant transformation via activating AKT/mTOR/4EBP1 signaling [92]. The multiple pro-stemness effects of CAFs on hepatic CSCs may provide promising potential targets for CSCs eradication therapy.

Apart from CAFs, ECs and adipocytes are essential elements in the tumor stroma. The vascular niche in TME generates instructive angiocrine factors to nourish CSCs [93]. It was reported that chemokine (C-X-C motif) ligand 9 (CXCL9) in the co-culture system of vascular ECs and CD133+ hepatic CSCs could enhance the migration and invasion of CD133+ cells with the activation of NF-kB signaling [94]. Moreover, lymphatic ECs could produce and secrete IL-17A to potentiate the self-renewal and tumorigenesis properties of hepatic CD133+ stem cells by upregulating programmed cell death-ligand 1 (PD-L1) expression [95]. Besides, adipocytes differentiated from mesenchymal stem cells (MSCs) have been found to secrete factors including IL-6, IL-8 and monocyte chemoattractant protein 1 (MCP1) to foster the CSC behaviors of CD133+EpCAM+ HCC cells via MET, STAT3 and ERK1/2 signaling [96].

In the context of the tumor immune environment, the immune cell subsets help to promote the tumor-initiating potential of liver CSCs. As the most studied immune cell type with an ineligible effect on hepatic CSC stemness regulation, M2 TAMs were validated to produce IL-6, which subsequently increased the CD44+ hepatic CSC proportion and tumorigenesis through the STAT3 signaling pathway [27]. TAMs could also augment the stemness characteristics of HCC cells by TGF-β-mediated EMT [97] and CC chemokine ligand 17 (CCL17)-induced EMT, TGF-β1 and Wnt/β-catenin signaling [98]. Besides, TAMs-derived TNF-α played critical roles in inducing the conversion of hepatic CSCs via the TNFR1/STAT3 pathway [99] and boosting EMT and stemness features via the Wnt/β-catenin pathway [100]. Interestingly, a recent study revealed that S100 calcium-binding protein A9 (S100A9) from TAMs improved the stem cell-like properties of HCC cells [101]. In addition, TANs in the TME are also critical inflammatory cells involved in liver cancer initiation, development, and progression. By co-culture with HCC cells, TANs were found to contribute essentially to tumor cell stemness by secretion of TGF-β2 and bone morphogenetic protein 2 (BMP2) to hyperactivate NF-κB signaling [102]. Regulatory T (Treg) cells, which are a subset of immune cells with pro-tumorigenesis capabilities, have also been recognized to exert potent effects on stemness properties of CSCs in HCC [103,104]. Tregs produced high levels of TGF-β1 to promote the generation of liver CSCs by mediating the EMT process [105]. It has been reported that the cancer autocrine molecule C-X-C motif chemokine 11 (CXCL11), accumulated in the TME, could increase the expression of stemness-related genes and maintain the stem cell features of α2δ1+ liver CSCs through chemokine (C-X-C motif) receptor 3 (CXCR3)/ERK1/2 signaling [106]. Simultaneously, CXCL11 could recruit activated T cells to infiltrate into the TME. However, the origins of CXCL11 and whether and how these recruited T cells support the CSC niche are still elusive. For other subtypes of T cells, such as CD8+ cytotoxic T cells, T helper cells Th1 and Th17, several studies have suggested that interferon gamma (IFN-γ) from CD8+ cytotoxic T cells and Th1 could promote the stemness traits in lung cancer [107]. Also, the IL-17 from Th17 was able to augment the self-renewal of CSCs depending on the STAT3 pathway in ovarian cancer [108], pancreatic cancer [109] and gastric cancer [110]. Nevertheless, the potential roles of these T cell subsets in regulating CSC stemness behaviors in HCC have been less explored.

With regard to other neighboring immune cells such as B cells and NK cells, CSCs impose a series of immuno-inhibitory effects on these cells to evade immunosurveillance [111,112,113]. Nevertheless, whether these cancer cell-recruited B cells and NK cells possess the capability to augment stemness in HCC needs to be further elucidated. The antigen-presenting cells-DCs present tumor antigens to cytotoxic immune cells and instigate an immune response. Cumulative studies have demonstrated that hepatic CSCs could alter the phenotypes of DCs to limit their effects on T cell activation and thus induce immunotolerance [114,115]. However, the reciprocal interactions between liver CSCs and DCs are still far from clear. More explorations are necessary to clarify their interplays and explicate how DCs modulate the stem cell-like characteristics of hepatic CSCs.

**Figure 2:**
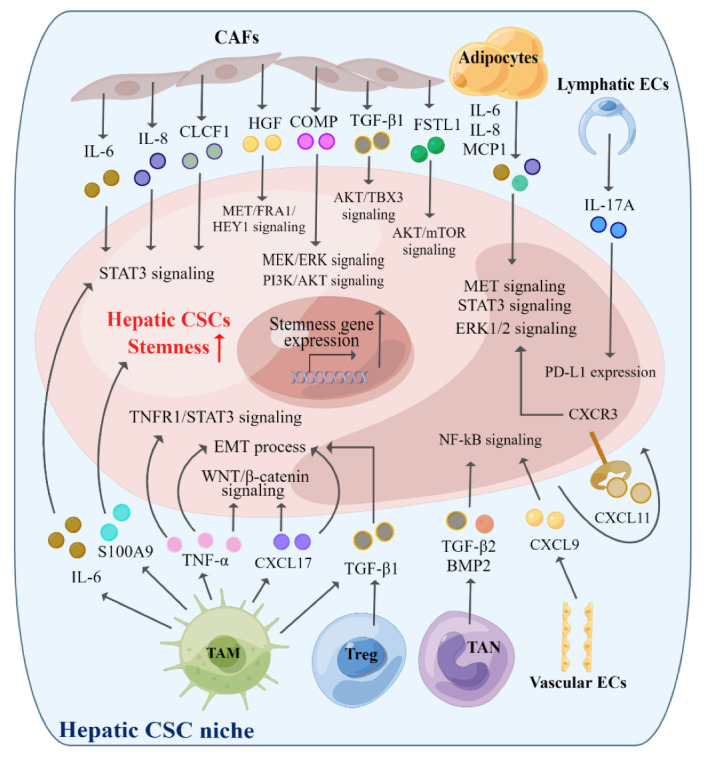
Cellular components potentiate stemness maintenance in hepatic CSCs. Cellular components in the TME include stroma cells such as cancer-associated fibroblasts (CAFs), adipocytes, endothelial cells (ECs) and various kinds of immune cells, including tumor-associated macrophages (TAMs), tumor-associated neutrophils (TANs), T cells, B cells, natural killer (NK), dendritic cells (DC) and myeloid-derived suppressor cells (MDSC). Among these cell subsets, CAFs, adipocytes, ECs, M2 TAMs, TANs and Tregs have been demonstrated to support the hepatic CSC niche. (By Figdraw.)

Additionally, MDSCs exert immunosuppressive and pro-tumor roles in the TME [116]. It was reported that HCC-derived IL-6 hindered the recruitment of MDSCs and enhanced their immune inhibitory function to hamper anti-tumor immunity [117]. Reciprocally, MDSCs-derived IL-6 conferred stemness properties to breast CSCs via STAT3 and NOTCH signaling pathways [118]. Nevertheless, how MDSCs mediate and maintain the stemness and malignant characteristics of liver CSCs has not come to light yet. Taken together, extrinsic cellular components in the tumor microenvironment play indispensable roles in the stemness modulation of HCC CSCs (Figure 2). Further investigation into the underlying mechanisms will provide a better understanding of the cellular crosstalk between CSCs and the TME and shed light on the development of new therapeutic strategies targeting CSCs.

## 4. CSCs-Targeted Therapy in HCC

Recent decades have witnessed rapid advancements in CSCs-targeted therapy. In the present review, we have focused on the intrinsic expression and identification of hepatic CSCs surface markers, the heterogeneity of marker-positive subpopulations, and elucidated how the extrinsic cellular elements in the TME colluded with hepatic CSCs to augment their stemness properties. Below, we synopsize the currently valuable hepatic CSCs-targeted therapeutic strategies through interference with these intrinsic and extrinsic molecular mechanisms, including targeting hepatic CSC surface markers and debilitating the cellular support for liver CSCs from the TME.

### 4.1. Targeting Surface Markers of Hepatic CSCs

Utilizing antibodies, inhibitors, or combinational techniques to target CSC surface markers can powerfully diminish hepatic CSCs. The administration of oncolytic viruses in alleviating tumors has been found as an emerging anti-cancer approach with specific cytotoxicity against cancer cells [119]. It was reported that CD133+ liver cancer cells could be eliminated explicitly by the CD133-targeted oncolytic measles virus termed MV-CD133 [120]. A recent study further revealed that the vesicular stomatitis virus targeting CD133 (VSV-CD133) exerted more powerful oncolytic and anti-tumor activity than MV-CD133 in HCC [121]. Furthermore, several antibody-based strategies can also target surface markers of CSCs, including direct inhibition using monoclonal antibodies and the indirect effect of antibody-induced cytotoxicity, such as the genetically engineered chimeric antigen receptor (CAR) T cells and dendritic cell vaccines. For example, an anti-CD3/anti-CD133 bispecific antibody binding to cytokine-induced killer cells (BsAb-CIK) was generated and exhibited a significant killing effect on CD133-high CSCs [122]. Recently, a clinical trial (NCT02541370) exploring the activity of CD133-guided CAR T cells in various cancers, including HCC, has been accomplished, showing the safety and efficacy of CD133-targeted CAR T-cells in HCC patients [123,124]. Another study tested the efficacy of the CD133-directed dendritic cell vaccine in HCC and demonstrated that this vaccine could induce CD8+ cytotoxic T cell activity against CD133+ liver CSCs [125]. CD44 and EpCAM peptide-loaded DCs vaccines were also evidenced to endow dramatic anti-tumor immunity to HCC cells [126]. Interestingly, the anti-CD44 antibody was reported to have an inhibitory effect on CD90+ CSCs in HCC cells by inducing apoptosis [77]. Besides, VB4-845, an inhibitor targeting EpCAM+ CSCs in HCC, showed effective anti-tumor cytotoxicity and synergistic effects in combination with 5-FU [127]. Currently, two ongoing clinical trials are evaluating the anti-tumor activity of EpCAM-based CAR T (NCT03013712, NCT02729493) in primary or refractory liver cancer. In addition, novel compounds targeting liver CSCs are also under development. The Kübra group reported that their newly synthesized isoxazole-piperazine analogue compounds could attenuate the proportion of CD133+EpCAM+ cells and downregulate pluripotency marker expression, implying their potential to be developed as CSCs-directed agents [128]. Similarly, the CD133+EpCAM+ cell population was discovered to be reduced by a novel nanoparticle of nitidine chloride named TPGS-FA/NC [129]. Furthermore, a known mTOR pathway inhibitor, OSU-CG5, could impede the maintenance of CD90+ CSCs in liver tumors [130].

Additionally, there are various inhibitors showing inhibitory effects on CD13+ CSCs. Bestatin (ubenimex), identified as a CD13 inhibitor, exerted a potent anti-tumor effect in preclinical mouse models and impeded the stemness features of liver CSCs [131,132]. Likewise, CD13+ CSCs could also be suppressed by a fusion protein, termed NGR-LDP-AE, composed of a CD13-targeted peptide NGR and anti-tumor antibiotic lidamycin, which displayed a prominent anti-tumor effect by killing CD13+ CSCs and suppressing angiogenesis [133]. Another novel conjugate termed poly (ethylene glycol)-poly (lysine) block copolymer-ubenimex conjugate (PEG-b-PLys (Ube)), which used the PEG as a delivery system, showed strong anti-tumor and synergistic effects with combinations of fluorouracil, cisplatin, or doxorubicin targeting CD13+ CSCs [134]. Surprisingly, the Zhou group recently identified a new organic selenium compound termed CU27 using a functional screening and found that CU27 displayed dramatic capacities to destem the CD13+CD133+ population by binding to c-MYC [135].

**Figure 3:**
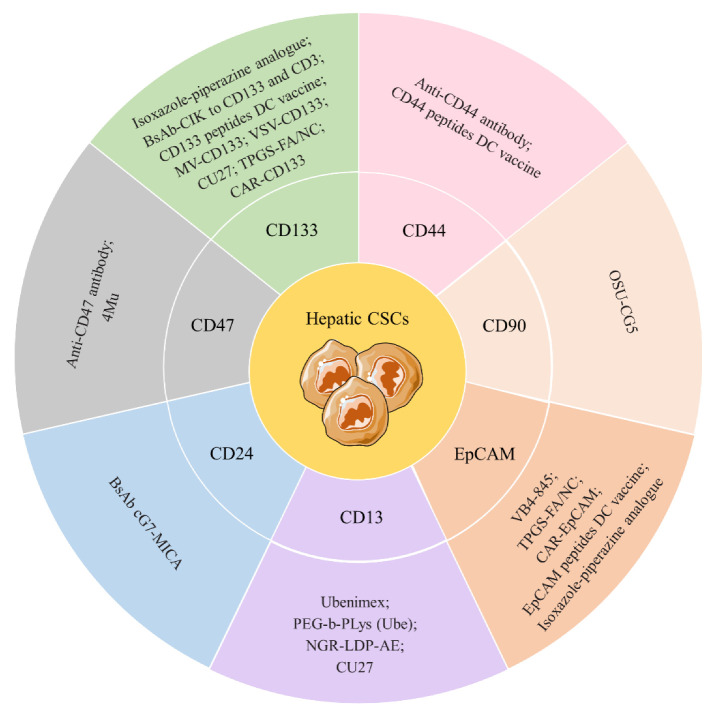
Current therapeutic strategies targeting hepatic CSC surface markers. The seven surface markers in different colored region are the essential stemness markers for hepatic CSCs. Distinct approaches, including oncolytic viruses, inhibitors, antibodies, CAR T cells therapy and vaccines, showed potent inhibitory effects on hepatic CSCs. Combining any of these therapeutic strategies may contribute to the better eradication of CSCs.

A BsAb was developed to synchronously recruit NK cells and target CD24+ hepatic CSCs, using the ligand of the NK cell receptor group 2 member D (NKG2D), the MHC class I-related chain A (MICA) to fuse with the flexible pentapeptide cG7 and form BsAb cG7-MICA [136]. It was stated that the hyaluronan synthesis inhibitor 4-methylumbelliferone (4Mu) could induce a potent anti-cancer efficacy in HCC by targeting the CD47+ CSCs [137]. Besides, applying an anti-CD47 antibody to block CD47+ CSCs in HCC significantly decreased stemness and sorafenib resistance [138]. A monoclonal antibody, 1B50-1 showed a therapeutic effect on HCC engraftments to eliminate CSCs by binding to α2δ1 [54]. The current therapeutic strategies targeting hepatic tumor CSC surface markers are shown in Figure 3. Given the complex heterogeneity of distinct marker-positive subpopulations in the CSC niche, novel strategies aiming to target more than one marker may be more successful in CSCs-directed therapy. Although the potential approaches directly targeting surface markers of liver CSCs have been demonstrated to be effective in eliminating CSCs, there is still a long way to go until their application in the clinic.

### 4.2. Hampering Cellular Supports for Hepatic CSCs in TME

As stroma cells and immune cells could confer pro-stemness capacities to hepatic CSCs through the secretion of various cytokines, chemokines, and secreted proteins (Figure 2), CSCs-based approaches impeding the supportive signals from TME would be promising to wipe out CSCs. Given that IL6 can be derived from various cell types, targeting IL-6 may exert potent CSCs-inhibitory effects. A previous study has illustrated that the blockade of IL-6 with tocilizumab, approved by the Food and Drug Administration for treatment of rheumatoid arthritis, could attenuate the self-renewal of CD44+ liver CSCs and decrease tumorigenesis, suggesting its potential to target liver CSCs and application in the clinical management of HCC patients [27]. A phase I clinical trial (NCT02536469) was completed, which tested the anti-tumor efficacy of humanized anti-IL-8 monoclonal antibodies in solid tumors, providing possibilities to utilize the IL-8 antibody to abrogate CSCs in HCC [139]. Besides, the production of CAFs-derived COMP could be diminished by a potential agent named resolvin D1 and thus obstruct the stemness properties of HCC CSCs [87]. In addition, the FSTL1 neutralizing antibody exerted significant effects on the eradication of the hepatic CSC subset and the reverse of sorafenib resistance [92]. Overall, the inhibitors or antibodies directly targeting the secreted proteins in the TME that contribute to stemness maintenance of liver CSCs are being discovered continuously. Further development of these agents may open a new era for CSCs-based therapy. The multiple hepatic CSCs-targeted therapeutic strategies are recapitulated in Table 2.

## 5. Conclusions

Hepatic CSCs remain the major culprit for intratumor heterogeneity, tumor aggressiveness, relapse, metastasis and resistance to chemotherapy and targeted therapy in HCC. The existence of CSCs poses great obstacles to the durative anti-cancer effect of multiple therapies. A profound understanding of the CSCs’ behaviors and their regulatory network will pave the way for developing novel and effective anti-CSCs therapeutic strategies. Numerous studies have demonstrated that the elements in the TME function as a supportive backup force to form a friendly niche for the maintenance of CSCs features. Our review gives an overview of the common and newly identified surface markers for the identification of hepatic CSCs as well as the impact of cellular components in the TME on liver CSCs. The heterogeneous expression patterns of distinct liver CSCs are also illustrated, which may provide novel directions for the development of CSCs-based therapy. Finally, we underline the currently practical CSCs-targeted therapeutic approaches based on the two avenues we summarized, in the hope of accelerating the development of CSCs-based therapy and ultimately improving HCC patient outcomes. The advancement in single-cell RNA-seq technology and 3D organoid culture of patient-derived tumors enables researchers to gain a comprehensive knowledge of hepatic CSCs in HCC tumors and investigate the bidirectional communications between CSCs and TME, facilitating our understanding of the CSC niche and further promoting translational medical research.

## Figures and Tables

**Table 2:** The multiple hepatic CSCs-targeted therapeutic strategies.

Approach	Agent or Inhibitor	Target	Applied Model	Refs.
Oncolytic viruses	MV-CD133 VSV-CD133	CD133	Cell lines Mouse Models	[119,120,121]
Antibody and cell-based	BsAb-CIK	CD133	Cell lines Mouse Models	[122]
CAR T	CAR-CD133	CD133	Clinical Trials	[123,124]
	CAR-EpCAM	EpCAM	Clinical Trials	/
Vaccines	DC vaccines	CD133 CD44 EpCAM	Cell lines Mouse Models	[125,126]
Compounds	VB4-845	EpCAM	Cell lines Mouse Models	[127]
	isoxazole-piperazine analogue	CD133 EpCAM	Cell lines	[128]
	TPGS-FA/NC	CD133 EpCAM	Cell lines Mouse Models	[129]
	OSU-CG5	CD90	Cell lines Mouse Models	[130]
	Bestatin	CD13	Cell lines	[131,132]
	NGR-LDP-AE	CD13	Cell lines Mouse Models	[133]
	PEG-b-PLys (Ube)	CD13	Cell lines Mouse Models	[134]
	CU27	CD133 CD13	Cell lines Mouse Models	[135]
	4Mu	CD47	Cell lines Mouse Models	[137]
	Resolvin D1	CAFs-derived COMP	Cell lines Mouse Models	[87]
Antibodies	anti-CD44 antibody	CD44 CD90	Cell lines Mouse Models	[77]
	BsAb cG7-MICA	CD24	Cell lines Mouse Models	[136]
	anti-CD47 antibody	CD47	Cell lines Mouse Models	[138]
	1B50-1	α2δ1	Cell lines Mouse Models	[54]
	Tocilizumab	CD44	Cell lines Mouse Models	[27]
	FSTL1 neutralizing antibody	CAFs-derived FSTL1	Cell lines Mouse Models	[92]

## Data Availability

Not applicable.

## Abbreviations

HCC, hepatocellular carcinoma; CSCs, cancer stem cells; TME, tumor microenvironment; PROM1, prominin 1; EpCAM, epithelial cell adhesion molecule; ICAM1, intercellular cell adhesion molecule 1; LGR5, leucine rich repeat containing G protein-coupled receptor 5; DLK1, delta-like 1 protein; CAFs, cancer-associated fibroblasts; ECs, endothelial cells; TAMs, tumor-associated macrophages; TANs, tumor-associated neutrophils; NK, natural killer; DCs, dendritic cells; MDSCs, myeloid-derived suppressor cells; HGF, hepatocyte growth factor; COMP, cartilage oligomeric matrix protein; EMT, epithelial–mesenchymal transition; CLCF1, cardiotrophin-like cytokine factor 1; CXCL6, chemokine (C-X-C motif) ligand 6; FSTL1, follistatin-like 1; CXCL9, chemokine (C-X-C motif) ligand 9; PD-L1: programmed cell death ligand 1; MSCs, mesenchymal stem cells; MCP1, monocyte chemoattractant protein 1; CCL17, CC chemokine ligand 17; S100A9, S100 calcium-binding protein A9; BMP2, bone morphogenetic protein 2; Regulatory T, Treg; CXCL11, C-X-C motif chemokine 11; CXCR3, chemokine (C-X-C motif) receptor 3; IFN-γ, interferon gamma; VSV-CD133, vesicular stomatitis virus targeting CD133; CAR, chimeric antigen receptor; BsAb, bispecific antibody; BsAb-CIK, bispecific antibody binding to cytokine-induced killer cells; PEG-b-PLys (Ube), poly (ethylene glycol)-poly (lysine) block copolymer-ubenimex conjugate; NKG2D, NK cell receptor group 2 member D; MICA, MHC class I-related chain A; 4Mu, 4-methylumbelliferone.
